# Length of stay in the emergency department and its associated factors among pediatric patients attending Wolaita Sodo University Teaching and Referral Hospital, Southern, Ethiopia

**DOI:** 10.1186/s12873-022-00740-3

**Published:** 2022-12-13

**Authors:** Kiberealeme Bisete Negasi, Almaz Tefera Gonete, Migbaru Getachew, Nega Tezera Assimamaw, Bewuketu Terefe

**Affiliations:** 1 School of Nursing Department of pediatrics and neonatology, Wolaita Sodo University, Wolaita Sodo, Ethiopia; 2 Department of Pediatrics and Child Health Nursing, School of Nursing, College of Medicine and Health Sciences, University of Gondar, Gondar, Ethiopia; 3 School of Nursing Department of emergency and critical care nursing, Wolaita Sodo University, Wolaita Sodo, Ethiopia; 4 Department of Community Health Nursing, School of Nursing, College of Medicine and Health Sciences, University of Gondar, Gondar, Ethiopia

**Keywords:** Ethiopia, Length of stay, Pediatrics emergency, Wolaita

## Abstract

**Background:**

Globally, there is an increase in the need for emergency department visits, which is exceptionally high in pediatric patients, resulting in longer lengths of stay, which is a global challenge and a hospital bottleneck that increases the risk of patient morbidity and mortality while also lowering satisfaction.

**Objective:**

This study aimed to assess the length of stay and associated factors in the pediatric emergency department at Wolaita Sodo University Hospital in 2021.

**Methods:**

An institution-based cross-sectional investigation was undertaken from March 15 to May 15, 2021. The 422 study participants were chosen using a systematic sampling procedure. The data were collected using semi-structured interviewer-administered questionnaires and chart reviews. Epi Data version 4.6 was used to enter the data, while SPSS version 26 was used to analyze it. With a 95% confidence interval, descriptive statistics were used to describe the prevalence, pediatrics, and emergency department duration of stay. The factors related to the length of stay were identified using bivariable and multivariable logistic regression analysis. On the AOR, a significant level was proclaimed when the *p*-value was less than 0.05, and the confidence interval was less than 95%.

**Results:**

The proportion of prolonged pediatric emergency department length of stay was 79.70% (95% CI; 75.7, 83.6). Nighttime arrival [AOR = 3.19, 95% CI (1.14, 8.98)], weekend arrival [AOR = 4.25, 95% CI (1.49, 5.35)], not receiving ordered medication in the hospital [AOR = 2.05, 95% CI (1.04, 4.03)], orange triage category [AOR = 4.01, 95% CI (1.60, 10.05)], and duration of pain 13–24 h [AOR = 0.29, 95% CI (0.89,0.98)], were significantly associated with length of stay.

**Conclusion:**

The percentage of children who stayed in the pediatric emergency department for an extended period was high. Policymakers should implement evidence-based care, maximize existing resources, provide equal access to care and high-quality care, and make pediatric emergency departments more accessible and operationally efficient.

## Background

The time spent in a pediatric emergency department (ED) between arrival and discharge, admission, or referral to another health facility is referred to as the length of stay (LOS) [1]. The ED at the hospital is designed to assess and stabilize patients who require immediate medical attention. It must be accessible 24 h a day, and immediacy is crucial for the quality of ED care [2–4]. It has become increasingly important in the healthcare delivery system, and it is now the major point of entry for inpatient admissions, discharges, and referrals [5, 6]. Longer pediatric ED stays are a global problem with variable prevalence rates, such as 31.3% in Guangzhou, China, 25.7% in north Taiwan, and 16.4% in Calabar, Nigeria [7–9]. In the last 20 years, there has been a global mismatch between emergency service supply and demand, with annual ED visits increasing faster than the growing population, resulting in more ED visits and longer ED stays [10, 11].

Due to an increased risk of hospital-acquired infections, morbidity, and death, as well as reduced patient satisfaction, length of stay (LOS) is associated to lower care quality, as well as parents’ socioeconomic concerns, hospital occupancy rates, more significant healthcare resource limits, and patient safety [6, 12–14]. Several studies have found that remaining in the emergency department for an extended period of time increases death rates by 15–30% and reflects poor hospital performance. It’s also a risk factor for pneumonia, with each hour raising the risk by around 20%; coping with this and other long-term effects is a worldwide problem [15–17]. Shorter hospital stays also save money by lowering unnecessary medical costs, boosting bed turnover, enhancing the institution’s profit margin, and lowering social-related charges [5, 9, 18].

Even though, LOS varies, several factors lead to lengthier pediatric ED stays. The most common predisposing factors for prolonged pediatric ED length of stay among these limited health care system capacities are professional inability to correctly triage, lack of decision, utilization of health care such as imaging or laboratory investigations, and increased flow of non-urgent cases to EDs [5, 19, 20]. As a result, infectious and preventable diseases such as malaria, diarrhea, and pneumonia are common in Sub-Saharan African countries, accounting for 70% of pediatric emergency admissions; this, combined with the rising prevalence of non-communicable diseases, may lengthen the time spent in pediatric emergency departments [9, 21]. In most low- and middle-income nations, the pediatric emergency specialization is underdeveloped. With minimal training in pediatric emergency medicine, a general doctor provides pediatric emergency unit treatment. As a result, there are important gaps in pediatric emergency treatment, which could have a severe impact on patient outcomes; this scenario is exacerbated in Ethiopia, where the ratio of doctors to population is low. In the event of an emergency, there is no pediatrician on call [9, 22].

Overcrowding in emergency department is a global concern representing a global crisis that might affect service quality due to the length of stay. Overcrowding is also a result of prolonged LOS [9, 23, 24]. Emergency triage assessment and treatment (ETAT) for health care personnel were being developed by world health organization (WHO) and Ethiopian federal ministry of health as potential approaches to reduce pediatric emergency department duration. Prolonged pediatric ED stays, on the other hand, remain a global problem [14]. In Ethiopia, a modest study was undertaken on characteristics associated with LOS in pediatric emergency departments (PED). Children must be highlighted because they represent a country’s future and demand special attention, particularly in terms of their health and access to high-quality care [18].

Longer pediatric ED stays are a worldwide problem that puts patients at risk for increased morbidity, death, lower satisfaction, increased financial burden on parents, and a reduction in healthcare resources. Overcrowding can occur as a result of longer LOS, and ED overcrowding is a global concern that implies a global crisis compromising service quality. ED LOS is an important part of quality assurance monitoring since it is linked to increased patient morbidity, mortality, and dissatisfaction. Children are vulnerable to hazards, and the longer they stay in the emergency department, the greater the risk; hence, identifying potential variables for LOS will aid in developing a tailored intervention to avoid prolonged LOS.

In Ethiopia, there is a scarcity of data on ED LOS. As a result, the findings of this study will give a snapshot of pediatric ED length of stay planning, policy-making, and ED interventions to address concerns associated with prolonged PED stays. Furthermore, this research will aid in the achievement of the Sustainable Development Goals in particular (SDG3). In addition, this study will look into variables such as parents’ work, marital status, prior treatment, and educational status, which have not been looked into in previous studies. As a result, fresh information on the LOS association in pediatric emergency departments will be reviewed in this study. Furthermore, the findings will raise awareness of the problem within the hospital and aid in the formulation of appropriate strategies to reduce LOS in the pediatric emergency department. Furthermore, the study’s findings will aid in the fight against lengthy LOS.

## Methods

### Study design and setting

An institutional-based cross-sectional study was conducted at Wolaita Sodo University Teaching Referral Hospital in Wolaita Sodo town from March 15 to May 15, 2021. Wolaita Sodo is 313 km from Addis Ababa, Ethiopia’s capital. It is one of the ten kebeles in Ethiopia’s Southern Region that make up the zonal administrative divisions of cities. There were 4963 women between the ages of 15 and 49, 28,499 children under the age of five, and 4576 infants under the age of one year in the reproductive age group. In the town, there are two hospitals (one public and one private), three health facilities, 19 medium and lower level clinics, and 17 health posts [25, 26].

Wolaita Sodo University Tertiary Referral Hospital (WSUTRH) was founded in 2007 and now offers outpatient and inpatient medical, surgical, pediatric, psychiatric, ophthalmology, emergency, gynecological, and obstetric services. The hospital serves more than three million people in Wolaita and neighboring zones, with an average bed capacity of 416 and roughly 1155 health professionals and other technical and other personnel [27].

The pediatric emergency admission unit, the Neonatal Intensive Care Unit (NICU), the pediatric surgical admission ward, the pediatric medical admission ward, and the stabilization center (SC) unit are the four main wings of the pediatrics department, which have a total of 133 patient beds. There are 29 beds in the pediatric emergency department [27].

### Participants of the study

All children who visited Wolaita Sodo University Hospital’s pediatric emergency department were included in the study’s source population. Children who visited a pediatric emergency department during the data collecting period were included in the study. Children who died shortly after arriving and patients who were critically ill were, on the other hand, omitted from the study.

### Sample size determination and sampling procedure

The sample size was calculated using a single population proportion formula based on the following statistical assumptions: *P* = 50% (assuming a 50% proportion of pediatric emergency department length of stay because there is no published study on the study area of interest), 95% confidence level, and a 5% margin of error. The sample size was calculated using this formula, and a 10% non-response rate was applied. As a consequence, data from 422 pediatric patients who visited the pediatric emergency department was acquired. The actual study participants were chosen using a rigorous random sample technique at Wolaita Sodo University’s pediatric emergency department. The average number of pediatric patients who visited Wolaita Sodo University Teaching and Referral Hospital’s pediatric emergency department in the three months prior to the study was determined using client registration to select the desired sample of pediatric patients among Wolaita Sodo University Teaching and Referral Hospital’s attendants. The estimated client flow rate for the research period (one month) was 1,200 based on this. After that, the sample interval (k) was computed by dividing the expected number of mothers visiting the unit throughout the study period (N) by the number of respondents (n) (1200/422 = 2.8). Finally, a systematic random selection process was used to select one out of every three attendant child combinations until the required sample was reached.

### Data collection tools and procedures

To obtain the necessary data, face-to-face interviews, semi-structured questionnaires, and chart reviews were employed. Tools were adapted and modified from previous studies [5, 10, 14, 22, 28, 29]. The questionnaire is divided into four sections. The first section contains 13 questions about the study participants’ socio-demographic characteristics. The second section includes five questions about time factors affecting the length of stay. The third section contains 11 questions about clinical aspects of the participant acting ED length of stay. The fourth section has 16 questions about organizational variables.

The data was collected thanks to the training of five nurses for data collection and one nurse for supervision, all of whom are Amharic and Wolaitigna native speakers. A pretest was provided to the twenty-one respondents at the Arbaminch referral hospital. The inter-observer variability was also investigated, and a correction based on the pretest results was used. The language fluency of the study participants was examined, and they were offered the choice of Wolaitigna or Amharic interviews. After confirming their willingness to engage in the study, verbal agreement and assent were obtained. Data was obtained from patients, and their parents via interviewer administered based questionnaire, and medical record inspections by qualified data collectors following their presentation, admission, and discharge from the ED.

The data gathering technique is depicted in the diagram below: First, data collectors in the triage department identified eligible patients, while resident personnel triaged patients as they came. As a result, when the patient entered the triage room, the time and other presentation factors were logged. Following that, at various treatment stages, additional information such as socio-demographic factors and other organizational-related elements was obtained through interviews and chart review. Once the patient was stabilized, diagnostic investigations and comprehensive treatment data were eventually recorded from medical records. The patient’s overall length of stay and final disposition, on the other hand, were documented as soon as the patient was discharged from the emergency department. Later, the essential time-related factors were ED arrival time and the overall length of stay between presentation and discharge from ED.

### Operational definitions

Pediatrics emergency department length of stay (PEDLOS): According to the Ethiopian federal minister of health, the duration between ED arrival and ED discharge, hospitalization, or referral to another health facility should not exceed 24 h [28].

Prolonged length of stay: A patient in the emergency department for more than 24 h is characterized as [28].

Waiting time: is a period that begins when a patient arrives at the ED and ends when the patient is triaged by health care providers and should not exceed 5 min [29].

### Data processing and analysis

Data were coded and put into Epi Data version 4.6 software after being checked for completeness, and then exported to SPSS version 26.0 for additional data analysis. Essential variables were provided in percent frequency tables and figures, as well as descriptive statistical analyses. Outliers and multicollinearity were checked in standardized residuals using the variance inflation factor, and variables with a variance inflation factor of more than ten were excluded. The Hosmer-Lemeshow goodness of fit test was used to assess model fitness.

To adjust for all possible confounders and identify the essential factors, independent variables with a *p*-value of 0.25 in the bivariable logistic regression analysis were included in the analysis. In the multivariable logistic regression adjusted odds ratios with 95% confidence intervals were used to assess the strength of the relationship between the dependent and independent variables with a *p*-value of < 0.05.

## Results

### Socio-demographic characteristics of the respondents

Four hundred twenty-two pediatric patient-attendant couples participated in the study, with 408 (96.68%) of them responding. The study participants’ median age was 2.06 years, with an Inter-Quartile Range (IQR) of 0.66–6.23 years. More than a third (40.4%) of them were under one year. Moreover, half of the study participants (55.4%) were female, and two hundred thirty-three (57.1%) lived in cities (Table 1).

**Table 1:** Socio-demographic characteristics of respondents at the pediatric emergency departments in Wolaita Sodo University Teaching and Referral Hospital, South Ethiopia, Jun 2021. (*n* = 408)

Socio-demographic characteristics	Length of stay		*P*-value
	Prolonged **n (%)**	Not prolonged **n (%)**	
**Age**			
≤1	135(80.4%)	33(19.6%)	0.333
1.1-5.0	97(80.2%)	24(19.8%)	0.362
5.1–12.0	72(80.0%)	18(20.0%)	0.392
≥12.1	21(72.4%)	8(27.6%)	Reference
**Sex**			
Male	143(78.6%)	39(21.4%)	0.625
Female	182(80.5%)	44(19.5%)	Refe.
**Residence**			
Rural	149(85.6%)	25(14.4%)	0.011
Urban	176(75.2%)	58(24.8%)	Refe.
**Distance to hospital in km**			
≤ 7 km	102(71.3%)	41(28.7%)	Refe.
8–25 km	41(77.4%)	12(22.6%)	0.004
> 26 km	182(85.5%)	30(14.2%)	0.001
**Religion**			
Orthodox	148(81.5%)	33(18.5%)	Refe.
Muslim	19(57.6%)	14(42.4%)	0.003
Protestant	103(76.9%)	31(23.1%)	0.321
^a^Other^1^	58(92.1%)	5(7.9%)	0.054
**Marital status**			
Single	70(71.4%)	28(28.6%)	0.214
Married	227(82.2%)	49(17.8%)	0.988
^b^others^1^	28(82.4%)	6(17.6%)	Refe.
**Educational status**			
No formal education	146(79.8%)	37(20.2%)	0.955
Attending formal education	179(79.6%)	46(20.4%)	Refe.
**Formal education level**			
Primary	77(82.8%)	16(17.2%)	0.792
Secondary	37(71.2%)	15(28.8%)	0.179
Above	65(81.2%)	15(18.8%)	Refe.
**Occupation**			
Governmental	72(77.4%)	21(22.6%)	Refe.
Private	90(82.6%)	19(17.4%)	0.361
Farmer	132(79.5%)	34(20.5%)	0.692
Others^2^	31(77.5%)	9(22.5%)	0.992
**Language spoken**			
One	89(85.6%)	15(14.4%)	0.698
Two	207(77.0%)	62(23.0%)	0.432
Three or more	29(82.9%)	6(17.1%)	refe
**Monthly income in ETB**			
≤ 500	24(77.4%)	7(22.6%)	0.532
500–1000	27(67.5%)	13(32.5%)	0.034
1001–2000	37(77.1%)	11(22.9%)	0.419
> 2000	237(82.0%)	52(18.0%)	refe.

^a^others^1^: catholic, ^b^others^1^, refe: reference, *km* kilo meter, *ETB* Ethiopian birr

### 5.2 Clinical related characteristics of respondents

One hundred forty-five (35.5%) of the respondents (*n* = 408) reported having respiratory symptom concerns. Nearly two-thirds of the respondents (61.3%) were on high alert for conciseness. The majority, 319 (54.9%), had had prior therapy before arriving at the ED. 89 (21.1%) of the respondents said they had co-morbid illnesses. The most prevalent initial diagnosis was respiratory difficulties, and all respondents had the same end diagnosis as the original diagnosis (Table 2).

**Table 2:** Clinical related characteristics of respondents at the pediatric emergency departments in Wolaita Sodo University Teaching and Referral Hospital, South Ethiopia, 2021. (*n* = 408)

Presenting characteristics	Length of stay		*P*-value
	Prolonged **n (%)**	Not prolonged **n (%)**	
**Mode of arrival by**			
Taxi	125(73.5%)	45(26.5%)	Refer.
Ambulance	30(90.9%)	3(9.1%)	0.042
Public transport	143(82.2%)	31(17.8%)	0.054
^ a^Others^1^	27(87.1%)	4(12.9%)	0.115
**Referral status**			
Self	188(75.2%)	62(24.8%)	0.006
Others^1^	137(86.7%)	21(13.3%)	Refe.
**Chief compliant**			
RS	129(89.0%)	16(11.0%)	0.020
CVS	36(81.8%)	8(18.2%)	0.430
GI	62(71.3%)	25(28.7%)	0.642
CNS	24(61.5%)	15(38.5%)	0.180
Trauma	38(84.4%)	7(15.6%)	0.263
Fever	36(75.0%)	12(25.0%)	Refer.
**Prior treatment**			
Yes	254(79.6%)	65(20.4%)	Refe.
No	71(79.8%)	18(20.2%)	0.975
**Prior treatment place**			
Home Remedy	114(72.2%)	44(27.8%)	0.002
Health facility	132(86.8%)	20(13.2%)	0.128
^a^Other^2^	79(80.6%)	19(19.4%)	Refe.
**Preexisting comorbidity**			
Yes	83(93.3%)	6(6.7%)	0.001
No	242(75.9%)	77(24.1%)	Refe.
**Triage Category**			
Red	113(92.6%)	9(7.4%)	0.000
Orange	88(88.0%)	12(12.0%)	0.000
Yellow	78(69.6%)	34(30.4%)	0.290
Green	46(62.2%)	28(37.8%)	Refe.
**Mental status**			
Alert	164(73.2%)	60(26.8%)	Refe.
Comatose	50(87.7%)	7(12.3%)	0.026
Confused	111(87.4%)	16(12.6%)	0.002

Others^1^: health centers and hospitals

### 5.3 Time and related organizational factors of respondents

Nearly half (48.50%) of the total respondents (*n* = 408) arrived at the ED in the morning. Furthermore, most respondents (319, or 78.10%) went to the ED on workdays. All of the respondents had requested an inquiry, and all of them had laboratory tests, with 245 (60%) having imaging studies (Table 3).

**Table 3:** Organizational related factors of respondents’ characteristics at the pediatric emergency departments in Wolaita Sodo University Teaching and Referral Hospital, South Ethiopia, 2021. (*n* = 408)

Factors	Length of stay		*P*-value
	Prolonged **n (%)**	Not prolonged **n (%)**	
**Time of arrival**			
Morning	141(70.9%)	58(29.1%)	refe.
Afternoon	121(87.1%)	18(12.9%)	0.001
Night	90(63%)	7(10%)	0.002
**Day of arrival**			
Workday	242(76.1%)	76(23.9%)	refe.
Weekend	83(93.3%)	6(6.7%)	0.001
**Waiting time**			
≤ 5 min	147(176.6%)	45 (23.4%)	refe.
> 5 min	178(82.4%)	38(17.6%)	0.144
**Duration of pain**			
≤ 12 h	42(85.7%)	7(14.3%)	refe.
13–24 h	24(51.1%)	23(48.9%)	0.000
25–48 h	43(74.1%)	15(25.9%)	0.145
49–72 h	73(73.0%)	27(27%)	0.087
≥ 73 h	143(92.9%)	11(7.1%)	0.133
**Ordered investigations**			
Yes	325(79.7%)	83(20.3%)	
No	0	0	
**Laboratory test**			
Yes	325(79.7%)	83(20.3%)	
No	0	0	
**Imaging studies**			
Yes	216(88.2%)	29(11.8%)	refe.
No	109(66.9%)	54(33.1%)	0.000
**Got ordered medication in the hospital**			
Yes	179(73.7%)	64(26.3%)	refe.
No	148(88.4%)	19(11.6%)	0.000
**Number investigations**			
1	27(65.9%)	14(34.1%)	refe.

Reasons for not got the ordered investigation.

**Fig. 1:**
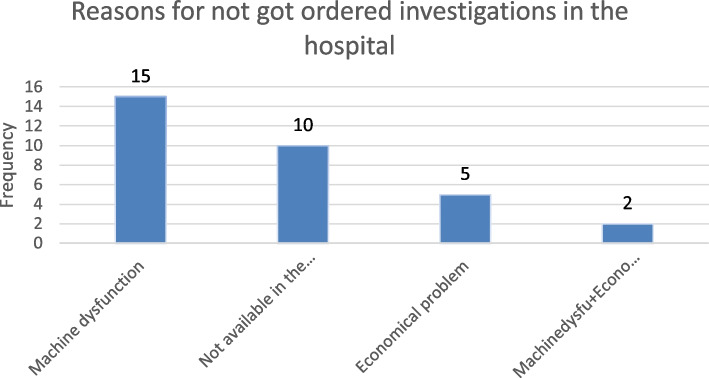
Reasons for not got the ordered investigation in pediatric emergency department of Wolaita Sodo University Teaching and Referral Hospital, South Ethiopia 2021. (*n* = 32)

Machine dysfunction was the common reason for not getting the order investigation (Fig. 1).

### The outcome of the respondents

**Fig. 2:**
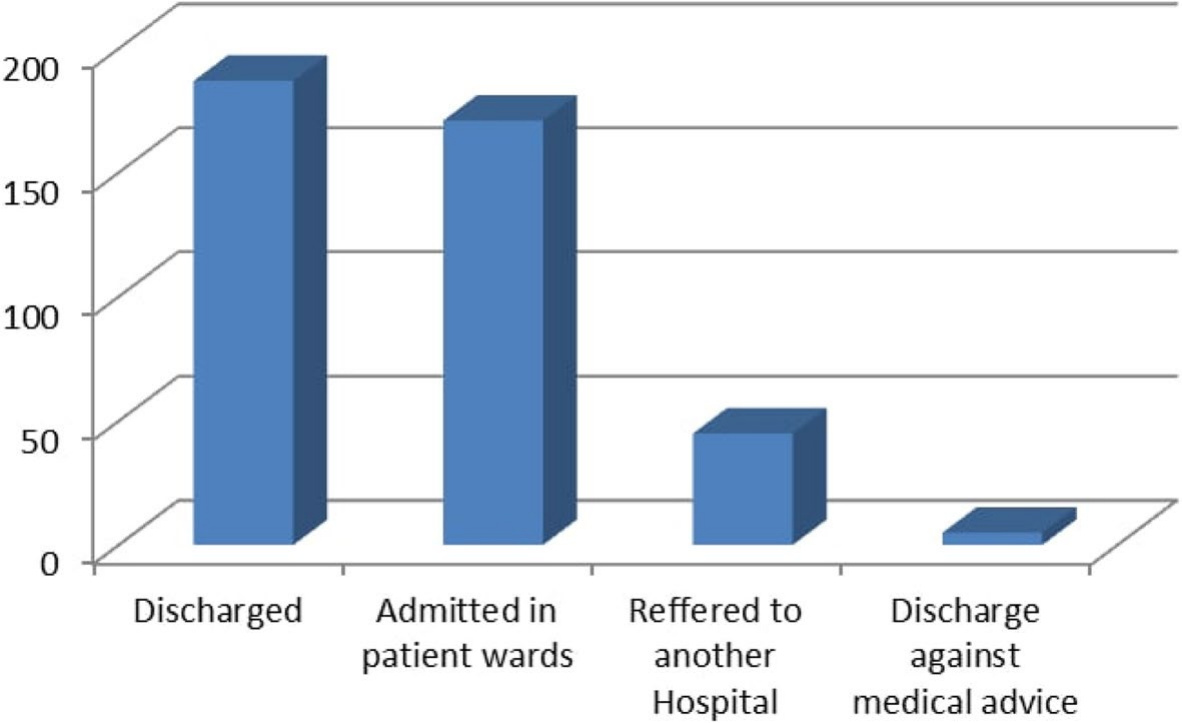
Outcome of pediatric patent from pediatrics emergency department of Wolaita Sodo University Teaching and Referral Hospital, South Ethiopia, 2021. (*n* = 408)

More than two-thirds of the responders (45.5%) were discharged from the pediatric emergency department due to physician orders. A small percentage of the participants (1.2%) were released against the medical recommendation. The least time taken from the time the physician ordered until the participants were discharged, admitted, and referred, respectively, was 2 min, 3 min, and 10 min. The maximum time taken to terminate was 60 min, 410 min to refer, and 417 min to admit (Fig. 2).

### Length of stay in the pediatric emergency department

**Fig. 3:**
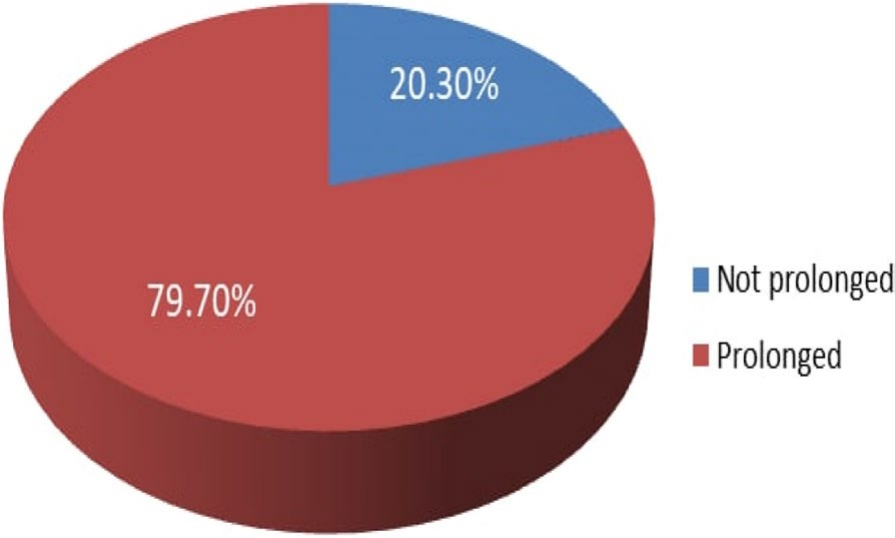
Prevalence of pediatrics emergency department length of stay in Wolaita Sodo University Teaching and Referral Hospital, South Ethiopia, 2021. (*n* = 408)

The number of respondents who had stayed in the pediatrics ED for more than 24 h was 325 (79.70%), whereas the rest, 83 (20.30%), had stayed for less than 24 h. The minimum length of stay in the pediatric emergency department of Wolaita Sodo University Teaching and Referral Hospital was 1 h, accounting for 1% of the participants, the median length of stay was 50.50 h, and the maximum ED length of stay was 384 h, accounting for 0.2% of the respondents (Fig. 3).

### Factors associated with length of stay

At a *P*-value of 0.2, the bivariable analysis revealed that residency, time of arrival, date of arrival, duration of pain, preexisting comorbidity, triage categories, imaging study, referral status, number of investigations, admission, and receiving the ordered medications in the hospital were all significantly associated with length of stay. Date of arrival, time of admission, duration of pain, triage categories, imaging study, and receiving all ordered drugs in the hospital all remained significant in the multivariable analysis.

Respondents who presented to the emergency department on weekends had 4.25 times (AOR = 4.25; 95% CI: 1.63, 11.12) higher odds of a more extended stay than those who contributed on weekdays. Patients who presented to the ED at night were 3.19 times (AOR = 3.19; 95% CI: 1.14, 8.98) more likely to have a protracted stay than those presented in the morning.

Those in the orange triage category were four times as likely than those in the green triage category to spend time in the PED (AOR = 4.01; 95% CI: 1.60, 10.05). Participants who did not locate all ordered medications in the hospital had 2.05 times (AOR = 2.05; 95% CI: 1.04, 4.03) higher chance of spending more time in the PED than those who did find all requested medications in the hospital. Those who had an imaging diagnostic investigation were 2.83 times (AOR, 2.82 95% CI, 1.49–5.35) more likely than those who did not have an imaging diagnostic investigation to spend a lengthy period in the PED.

Furthermore, the results of this study showed that the length of stay was 71% (AOR = 0.29; 95% CI: 0.87–0.98) less in a participant with a duration of pain from 13 to 24 h compared to those who had less than 12 h duration of pain (Table 4).

**Table 4:** Bivariate and Multivariable logistic regression analysis of factors associated with length of stay among pediatric patients attending pediatric ED of Wolaita Sodo University Teaching and referral hospital south Ethiopia, 2021 (*n* = 408)

Variables		Length of stay		COR (95% CI)	AOR (95% CI)	*P*-value
		**Prolonged N (%)**	**Not prolonged N (%)**			
**Residence**	Rural	149(85.10)	26(14.90)	1.86(1.12–3.09)	1.14(0.56–2.29)	0.724
	Urban	176(75.50)	57(24.50)	1.0	1.0	
**Time of arrival**	Morning	141(71.20)	57(28.80)	1.0	1.0	
	Afternoon	121(85.80)	20(14.20)	2.45(1.39–4.30)	1.83(0.92–3.69)	0.086
	Night	63(91.30)	6(8.70)	**4.24(1.73–10.35)**	**3.19(1.14–8.98)**	0.028*
**Date of arrival**	Working day	242(75.90)	77(24.10)	1.0	1.0	
	Weekend	83(93.30)	6(6.70)	**4.40(1.85–10.45)**	**4.25(1.63–11.12)**	0.003*
**Duration of symptoms or pain**	≤ 12 h	42(85.70)	7(14.30)	1.0	1.0	
	13–24 h	24(51.10)	23(48.90)	**0.17(0.65 − 0.46)**	**0.29(0.89–0.98)**	0.046*
	25–48 h	43(74.10)	15(25.90)	0.48(0.18–1.29)	0.69(0.20–2.37)	0.559
	49–72 h	73(73.000	27(27.00)	0.45(0.18–1.12)	1.09(0.35–3.45)	0.897
	≥ 73 h	143(92.90)	11(7.10)	2.17(0.79–5.94)	2.95(0.82–10.54)	0.096
**Preexisting comorbidity**	Yes	83(93.30)	6(6.70)	4.40(1.85–10.48)	1.98(0.73–5.30)	0.176
	No	242(75.90)	77(24.10)	1.0	1.0	
**Triage category**	Read	81(89.00)	10(11.00)	5.04(2.25–11.32)	2.51(0.87–7.23)	0.088
	Orange	120(90.90)	12(9.10)	**6.22(2.92–13.27)**	**4.01(1.60-10.05)**	0.003*
	Yellow	79(70.50)	33(29.50)	1.49(0.79–2.78)	1.35(0.63–2.88)	0.441
	Green	45(61.60)	28(38.40)	1.0		
**Imaging study**	Yes	216(88.20)	29(11.80)	**3.69(2.22–6.12)**	**2.82(1.49–5.35)**	0.001*
	No	109(66.90)	54(33.10)	1.0	1.0	
**Got all ordered medication**	Yes	179(73.70)	64(26.30)	1.0	1.0	
	No	145(88.40)	19(11.60)	**2.73(1.56–4.76)**	**2.05(1.04–4.03)**	0.037*
**Admit**	Yes	154(86.50)	24(13.50)	2.21(1.31–3.73)	1.46(0.78–2.74)	0.236
	No	171(74.30)	59(25.70)	1.0	1.0	
**Referral status**	Self	188(75.20)	62(24.80)	1.0	1.0	
	From other health institutions	137(86.70)	21(13.30)	2.15(1.25–3.69)	0.82(0.37–1.82)	0.624
**Number of investigations**	1	27(65.90)	14(34.10)	1.0	1.0	
	≥ 2	298(81.20)	69(18.80)	2.24(1.12–4.49)	1.28(0.52–3.13)	0.594

1.0=reference category, *=statistically significant at *P*-value <0.05

## Discussion

Patient length of stay is a significant indicator reflecting how well an ED is working and the overall efficiency and quality of service of an ED [30]. Longer lengths of stay are linked to higher rates of illness, mortality, and dissatisfaction; also, length of stay is a reasonable substitute for resource consumption and is critical for care planning and management [22].

In this study, 79.7% (95% CI: 75.7, 83.6) of pediatric patients stayed in the pediatric emergency department for more than 24 h. The high prevalence of prolonged pediatric emergency department stays a global issue, with 31.3%, 25.75, 22%, 10.2%, and 16.4% in Guangzhou, China, northern Taiwan, Boston, Iran, and Nigeria, respectively [4, 7–9, 31]. The disparity in the health care system level, developments in medical health services, and the variation in the number of health experts in Guangzhou, China, could be the cause of these variances. Five general physicians, 16 senior pediatric residents, and 22 nurses work in the emergency department [7], while according to the evidence found from the Wolaita Sodo University Teaching and Referral Hospital record, in the current study, the ED has four pediatricians, five general physicians, and seven nurses.

In addition, variations of criteria were used across settings; for instance, the study done in Nigeria used a greater ≥ 72 h for a prolonged length of stay [9]; nevertheless, the current study used > 24 h based on Ethiopian hospital services transformation guidelines criteria for the size of stay [32]. Those mentioned above might be the possible reason for an increased prevalence of prolonged pediatric ED length of stay in the current study.

The present study showed that nighttime arrival at PED was significantly associated with prolonged length of stay at PED. This follows other studies conducted in China, Indonesia, and the USA [3, 33, 34].

The possible explanation could be that the hospital may not admit inpatient or discharge patients during the night shift; night shift arrivals to ED need to wait until daytime to be accepted or discharged [32]. In addition, when compared to today, expert consultation and diagnostic or therapeutic modalities are less available at night. Morning arrival is a determinant for a prolonged length of stay in pediatric emergency departments, according to research conducted in Egypt and Switzerland [14, 35]. This disparity could be attributable to the fact that each hospital or region has its population features, arrival patterns, and staffing policy. This data can be helpful in planning providers at the administrative level.

Similarly, the outcomes of this study revealed that arriving at PED on the weekend was substantially associated with a more extended stay at PED. This outcome is consistent with findings from other Taiwanese and Chinese investigations [8, 36]. This could be because fewer health professionals are available on weekends, and experts (senior physicians, radiologists) may be unavailable on weekends compared to weekdays.

The triage category (orange) was found to be substantially related to a longer length of stay in this study. The same may be said for research undertaken in Indonesia, the United States, China, and Switzerland [3, 7, 14, 34]. This could be because patients on triage level II have a high level of comprehension that as the severity of the condition increases, the patients demand more care and emergency interventions take more time and extensive treatment. Similarly, the nature of the disease in the pediatric population is challenging to manage for health professionals, and the higher the acuity level, the more difficult it is for physicians to decide whether the patient should be admitted to an inpatient ward or referred to another hospital, or discharged. Furthermore, patients with high acute levels may require the assistance of experts (pediatricians) and additional inquiry methods. This may contribute to prolonged LOS by increasing the time to wait for the pediatrician and found ordered investigation.

This study also showed that having an ordered imaging study was significantly associated with prolonged PED length of stay; this is in line with a study conducted in Taiwan [34]. This could be that imaging investigations are required for accuracy and confirmation of the patient’s diagnosis, which is necessary for clinicians to decide whether the patient should be referred to another hospital, discharged, or admitted. Furthermore, most imaging investigations take longer than other investigations, which may result in a more extended stay in the pediatric emergency department.

This study also discovered that individuals who did not receive recommended drugs in the hospital had a considerably longer PED stay. The rationale could be that not getting the medicines in the hospital would result in more time spent looking for pharmaceuticals outside of the hospital, resulting in the late intake of the prescribed medication and a delay in recovery. Furthermore, the duration of pain had a substantial impact on the length of time spent on a PED, according to this study. PED length of stay was 71% less standard among patients who arrived with a pain course of 13–24 h compared to individuals who came with a pain course of 12 h. This could be because people with pain duration of 13–24 h are more likely to have minor life events that are easily managed. Similarly, less severe instances may not require intensive care, and diagnosis mode for confirmation of suspected cases to provide suitable therapies could be acceptable. This suggested that the less acute individuals had a shorter stay in the PED and were in pain for a shorter period of time.

### Limitations of the study

#### Limitation

Variables like duration of pain, time is taken from ordered by a physician until discharge, referred, and admission was prone to recall biased; to overcome this, the data were collected at the point of treatment.

## Conclusion

The proportion of children who spent a long time in the pediatric emergency department was found to be high in this study. The length of stay in the pediatric emergency department was found to be substantially associated with nighttime arrival, weekend day arrival, triage categories, duration of pain, imaging study, and receiving ordered medication in the hospital. As a result, effective interventions must be developed and executed, with a focus on the aforementioned linked factors.

## Data Availability

All data and materials relevant to this study are available from the corresponding author whenever required.

## Abbreviations

ED: Emergency Department
EM: Emergency Medicine
ETAT: Emergency Triage Assessment and Treatment
ETB: Ethiopian Birr
FDMOH: Federal Minister of Health
IMNCI: Integrated Management of Newborn and Childhood Illness
LMICs: Low and Middle-Income Countries
LOS: Length of Stay
PEDLOS: Pediatrics Emergency Department Length of Stay
PLOS: Prolonged Length of Stay
SPSS: Statistical Package for Social Sciences
WSUTRH: Wolaita Sodo University Teaching and Referral Hospital
